# Clinical IRAK4 deficiency caused by homozygosity for the novel *IRAK4* (c.1049delG, p.Gly350Glufs*15) variant

**DOI:** 10.1101/mcs.a005298

**Published:** 2020-06

**Authors:** Alicia Jia, Elliot James, Henry Y. Lu, Mehul Sharma, Bhavi P. Modi, Catherine M. Biggs, Kyla J. Hildebrand, Alanna Chomyn, Stephanie Erdle, Hasandeep Kular, Stuart E. Turvey

**Affiliations:** 1 Division of Allergy and Clinical Immunology, Department of Pediatrics, BC Children's Hospital, Vancouver, British Columbia V5Z 4H4, Canada;; 2 Department of Microbiology and Immunology, Vancouver, British Columbia V5Z 4H4, Canada;; 3 Experimental Medicine Program, Faculty of Medicine, Vancouver, British Columbia V5Z 4H4, Canada;; 4 Department of Medical Genetics, BC Children's Hospital Research Institute, The University of British Columbia, Vancouver, British Columbia V5Z 4H4, Canada

**Keywords:** decreased inflammatory response, recurrent infections in infancy and early childhood, recurrent systemic pyogenic infections

## Abstract

The innate immune system allows for rapid recognition of pathogens. Toll-like receptor (TLR) signaling is a key aspect of the innate immune response, and interleukin-1 receptor-associated kinase 4 (IRAK4) plays a vital role in the TLR signaling cascade. Each TLR recognizes a distinct set of pathogen-associated molecular patterns (PAMPs) that encompass conserved microbial components such as lipopolysaccharides and flagellin. Upon binding of PAMPs and TLR activation, TLR intracellular domains initiate the oligomerization of the myeloid differentiation primary response 88 (MyD88), IRAK1, IRAK2, and IRAK4 signaling platform known as the Myddosome complex while also triggering the Toll/IL-1R domain-containing adaptor-inducing IFN-β (TRIF)-dependent pathway. The Myddosome complex initiates signal transduction pathways enabling the activation of NF-κB and mitogen-activated protein kinase (MAPK) transcription factors and the subsequent production of inflammatory cytokines. Human IRAK4 deficiency is an autosomal recessive inborn error of immunity that classically presents with blunted or delayed inflammatory response to infection and susceptibility to a narrow spectrum of pyogenic bacteria, particularly *Streptococcus pneumoniae, Staphylococcus aureus, and Pseudomonas aeruginosa.* We describe a case of IRAK4 deficiency in an 11-mo-old boy with concurrent *S. pneumoniae* bacteremia and *S. aureus* cervical lymphadenitis with a blunted inflammatory response to invasive infection. Although initial clinical immune profiling was unremarkable, a high degree of suspicion for an innate immune defect prompted genetic sequencing. Genetic testing revealed a novel variant in the *IRAK4* gene (c.1049delG, p.(Gly350Glufs*15)) predicted to be likely pathogenic. Functional testing showed a loss of IRAK4 protein expression and abolished TLR signaling, confirming the pathogenicity of this novel IRAK4 variant.

## CASE PRESENTATION

An 11-mo-old previously healthy boy who was born at term following an uncomplicated pregnancy presented with a 1-mo history of left-sided cervical lymphadenopathy. He lacked typical features of an infection such as fever and localized erythema, warmth, and tenderness; thus, he was initially suspected of having a malignancy. An ultrasound revealed lymphadenopathy, abnormal lymph node architecture, and central node liquefaction consistent with lymph node necrosis. Fine needle aspirate from the left cervical node was culture-positive for methicillin-sensitive *Staphylococcus aureus* but showed no features of malignancy. On the evening following this procedure, the patient developed a fever to 39.2°C, and a blood culture was drawn that grew *Streptococcus pneumoniae* (also known as pneumococcus). Despite being bacteremic, the patient did not display the typical inflammatory response with laboratory investigations showing C-reactive protein of 12 mg/L (reference range < 10 mg/L), total white blood cell count of 10.4 × 10^9^/L (reference range 5.3–16.0 × 10^9^/L), lymphocyte count 6.23 × 10^9^/L (reference range 4.00–13.50 × 10^9^/L), and neutrophil count of 3.08 × 10^9^/L (reference range 1.00–8.50 × 10^9^/L).

The presence of invasive pneumococcal infection concurrently with *S. aureus* adenitis and diminished clinical and laboratory response to these invasive infections prompted a further workup for inborn errors of immunity, with a particular focus on Toll-like receptor (TLR) signaling defects. Clinical immune investigations at this point were reassuring, with normal values for quantitative serum immunoglobulin A, M, and E levels, dihydrorhodamine assay to quantify the neutrophil respiratory burst, complement activity as measured by the CH50, and lymphocyte subset (T, B, and natural killer [NK] cell) quantification by flow cytometry. Exceptions included an elevated immunoglobulin G to 10.8 g/L (reference range 4.0–8.3g/L) and elevated CD3 and CD4 T-cell numbers to 7.16 × 10^9^/L and 4.92 × 10^9^/L, respectively (reference range 1.60–6.70 × 10^9^/L and 1.00–4.60 × 10^9^/L, respectively) which were thought to reflect his underlying chronic infection.

**Figure 1.**
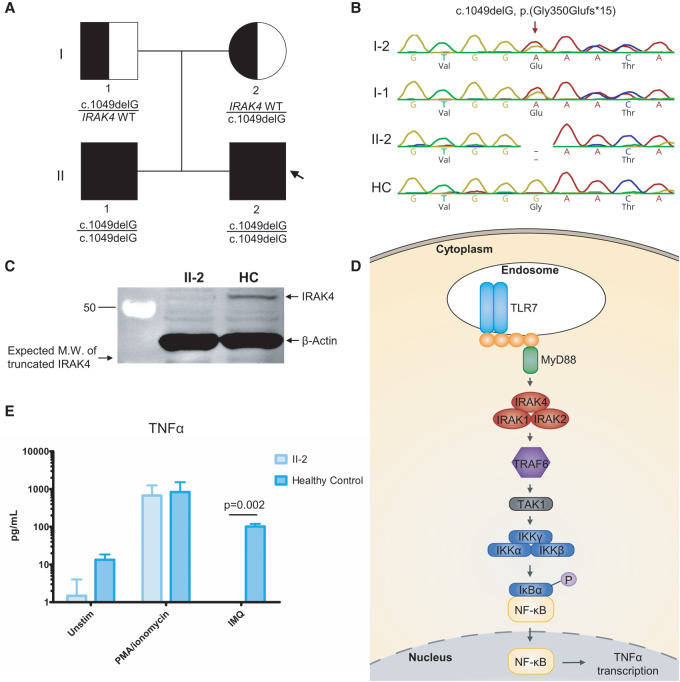
Pedigree and experimental analysis. (*A*) Pedigree depicting the presence of the c.1049delG, p.(Gly350Glufs*15) *IRAK4* variant. The arrow denotes the index patient. (*B*) Sanger sequencing of the *IRAK4* gene depicting the single-base-pair deletion in the affected family but not seen in the healthy control (HC). (*C*) Immunoblot showing lack of IRAK4 protein expression compared to a HC, as well as the predicted molecular weight (MW) and position of the truncated IRAK4 due to the variant (*n* = 3). (*D*) Simplified schematic overview of the TLR7 signaling pathway resulting in NF-κB activation and TNF-α transcription emphasizing the central role of the IRAK4 protein in signal transduction. (*E*) Cytometric bead array (CBA) showing no TNF-α response to imiquimod (IMQ) stimulation of TLR7 in the IRAK4-deficient cells compared to a HC. PMA/ionomycin serves as a positive control confirming cell viability. Data are represented as the mean (*n* = 3), and error bars represent the standard error of the mean (SEM). *P*-value calculated by the two-tailed Student's *t*-test with a Bonferroni correction.

Family history is significant for a brother aged 4 yr with a history of recurrent acute otitis media infections requiring oral antibiotics and one severe pneumonia requiring intravenous antibiotics at age 2 yr. The parents reported no consanguinity (Fig. 1A).

The patient's infections were treated with cefotaxime and the bacteremia resolved within 32 h; there were no further fevers. He completed a 2-wk course of intravenous cefotaxime followed by a 2-wk course of oral cephalexin. The left-sided cervical lymphadenitis persisted despite treatment. A repeat ultrasound after 3 wk off antibiotics revealed a large, thick-walled fluid collection with central debris in keeping with an abscess that was drained with fine needle aspiration. The culture did not grow any microorganisms, but 16S rRNA gene sequencing was positive for *S. aureus*. Intravenous cefazolin was initiated and follow-up 4 wk later revealed minimal interval change clinically or sonographically, so he was brought to the operating room for left neck dissection and lymph node debulking with incision and drainage of the abscess. The pus from the abscess was cultured but did not grow any microorganisms, and a 16S rRNA gene sequencing was negative. Surgical pathology from a piece of resected lymph node tissue revealed necrotizing granulomatous inflammation with no organisms identified. Cefazolin continued intravenously for another 4 wk totaling an 8-wk duration with near-complete resolution of the lymphadenitis.

Because of the high clinical index of suspicion for an underlying immune defect, genetic testing was pursued.

Tables 1 and 2 summarize key clinical and immunologic features.

**Table 1.** Clinical findings

IRAK-4 deficiency clinical findings^a^	Proband	Sibling (brother)	Mother	Father	Relevance/alternate explanation
Recurrent *Streptococcus pneumoniae* infections (HP:0005366)	Yes	Possible cause of severe pneumonia but not microbiologically confirmed	No	No	
Recurrent staphylococcal infections (HP:0007499)	Yes	No	No	No	
Absent or diminished signs of infection/inflammation	Yes	Unclear	No	No	Brother's pneumonia occurred out of country; fever was present but unclear if other signs of inflammation were diminished.
Autosomal recessive inheritance	Yes	Yes	No	No	Mother and father both healthy carriers of the variant.
Vaccination status (including pneumococcal vaccine)	Fully immunized	Fully immunized	Unknown	Unknown	

^a^List of clinical features include relevant Human Phenotype Ontology (HPO) terms.

**Table 2.** Laboratory findings

IRAK4 deficiency immunologic findings	Proband result	Age-specific reference range	Relevance and interpretation
White blood cell count	10.4 × 10^9^/L	5.3–16.0 × 10^9^/L	Inappropriately low as measured in the context of severe bacterial infection
Neutrophil count	3.08 × 10^9^/L	1.00–8.50 × 10^9^/L	Inappropriately low as measured in the context of severe bacterial infection
Lymphocyte count	6.23 × 10^9^/L	4.00–13.50 × 10^9^/L	Normal result
C-reactive protein	12 mg/L	<10 mg/L	Inappropriately low as measured in the context of severe bacterial infection
CD3 T-cell count (absolute)	7.16 × 10^9^/L	1.60–6.70 × 10^9^/L	Elevated, likely a reflection of underlying chronic infection
CD4 T-cell count (absolute)	4.92 × 10^9^/L	1.00–4.60 × 10^9^/L	Elevated, likely a reflection of underlying chronic infection
CD8 T-cell count (absolute)	1.84 × 10^9^/L	0.40–2.10 × 10^9^/L	Normal result
CD19 B-cell count (absolute)	1.54 × 10^9^/L	0.60–2.70 × 10^9^/L	Normal result
CD3-56^+^ NK-cell count (absolute)	0.27 × 10^9^/L	0.17–1.10 × 10^9^/L	Normal result
Hemolytic complement CH50	98%	>68%	Normal result
Dihydrorhodamine assay stimulated	1.00	0.90–1.00	Normal result
IgG	10.8g/L	4.0–8.3g/L	Elevated, likely a reflection of underlying chronic infection
IgM	0.25g/L	0.08–0.8g/L	Normal result
IgA	1.24g/L	0.06–1.45g/L	Normal result
*Corynebacterium diphtheriae* toxin antibody	0.06 IU/mL	N/A	Uncertain protection, indicates uncertain response to vaccine
*Clostridium tetani* toxin antibody	0.12 IU/mL	N/A	Immunity present, indicates adequate immune response to vaccine
Hepatitis B surface antibody	31.3 IU/mL	N/A	Immunity present, indicates adequate immune response to vaccine

## TECHNICAL GENETIC ANALYSIS

A primary immunodeficiency gene panel assessing 274 genes implicated in inborn errors of immunity was performed using a next-generation sequencing platform through a clinically accredited genetic testing company. Testing revealed that the proband was homozygous for a novel frameshift variant in *IRAK4* (OMIM:606883; c.1049delG, p.(Gly350Glufs*15). The variant results in a 1-bp deletion in exon 9, generating a frameshift predicted to lead to a premature stop codon at position 15 downstream in the new reading frame. The variant has not been observed in gnomAD (Genome Aggregation Database) and was predicted to cause loss of normal protein function. Based on the available evidence (mutation type, predicted impact, absence in large control population, and the genotype–phenotype correlation), the variant was classified as “Likely Pathogenic” at the time of reporting.

Table 3 summarizes the genetic findings.

**Table 3.** Genetic findings

Gene	Genomic location (GRCh37/hg19 coordinates)	HGVS cDNA	HGVS protein	Zygosity	Parent of origin	Variant interpretation
*IRAK4*	Chr 12: 44176217	NM_016123.3: c.1049delG	p.(Gly350Glufs*15)	Homozygous	Maternal and paternal	Pathogenic based on functional data presented in this report. Supporting evidence for pathogenicity criteria: PVS1, PS3, PM2, and PP1.

## VARIANT INTERPRETATION and FUNCTIONAL TESTING

Follow-up familial genetic testing revealed that both parents were heterozygous for the variant and the older brother was also homozygous for the *IRAK4* c.1049delG, p.(Gly350Glufs*15) variant (Fig. 1A,B).

Immunoblotting for the IRAK4 protein confirmed the absence of IRAK4 protein in patient lymphoblastoid cell lines (LCLs) compared to a healthy control, likely because of nonsense-mediated mRNA decay (Fig. 1C). To investigate the functional impact of the loss of IRAK4 protein, we assessed the function of the TLR signaling cascade. As a positive control, we found that nonspecific chemical activation of the cells with phorbol 12-myristate 13-acetate (PMA) and ionomycin resulted in comparable levels of tumor necrosis factor-α (TNF-α) production in both IRAK4-deficient and healthy control LCLs. However, following stimulation with imiquimod (IMQ), which is a specific activator of TLR7, there was no TNF-α produced by the IRAK4-deficient LCLs (Fig. 1E).

Subsequent variant interpretation according to the ACMG/AMP guidelines (Richards et al. 2015) indicates that the variant can be classified as “Pathogenic,” with supporting evidence for pathogenicity criteria PVS1, PS3, PM2, and PP1. We have thus confirmed the pathogenicity of the *IRAK4* c.1049delG, p.(Gly350Glufs*15) variant by demonstrating that this variant abrogated IRAK4 protein expression and abolishes signaling through TLR7 that utilizes IRAK4 as a signaling intermediate.

## SUMMARY

We have described a novel variant causing the human inborn error of immunity, IRAK4 deficiency (OMIM #607676). IRAK4 deficiency specifically abrogates TLR signaling through every known TLR with the exception of TLR3 (Turvey and Broide 2010). Impaired TLR signaling results in a blunted inflammatory response as the immune system fails to recognize and contain the infection. Patients with IRAK4 deficiency are at risk of potentially fatal bacterial infections in infancy and childhood. IRAK4 deficiency was first described in 2003 and has an estimated mortality rate of 40% (Picard et al. 2003, 2010; Yang et al. 2005). Patients with IRAK4 deficiency usually present with recurrent, invasive bacterial infections. *S. pneumoniae* is the most common cause of invasive infections in these patients, followed by *S. aureus* and *Pseudomonas aeruginosa* (Grazioli et al. 2016; Gobin et al. 2017). These patients appear to have normal immunity to viral, fungal, and parasitic infections. There is a reduced susceptibility to bacterial infections when children with IRAK4 deficiency reach school age. There is no experimental data explaining the observed immunological “improvement,” but the development of a more effective adaptive immune response is widely attributed to compensate and help protect these patients (Puel et al. 2004).

Immunological evaluation in IRAK4 deficiency usually reveals intact leukocyte populations, including T-, B-, and NK-cell numbers, as well as T cells that exhibit normal proliferation. Serum immunoglobulin values and antibodies against specific vaccine antigens are usually within the normal range with the exception of possible impaired responses to polysaccharide vaccines. The diagnosis of IRAK4 deficiency can be difficult and requires a high index of suspicion, as there may be no indicators on routine immunological evaluation. IRAK4 deficiency is generally diagnosed with a combination of genetic testing and functional studies showing impaired cytokine response to most TLR ligands (Picard et al. 2003, 2010; Davidson et al. 2006; Hirschfeld et al. 2007). Treatment includes optimization of pneumococcal vaccination status, antibiotic prophylaxis against *S. pneumoniae* ± *S. aureus*, and potentially the use of immunoglobulin replacement (e.g., IVIG). Duration of antimicrobial prophylaxis ± IVIG is also a point of controversy. Most experts agree to treat until at least the teenage years when the susceptibility to infection seems to be reduced, although others recommend lifelong treatment given the fact that the genetic failure of TLR signaling does not improve with age.

Our demonstration that the novel *IRAK4* c.1049delG, p.(Gly350Glufs*15) variant abrogates IRAK4 protein expression and abolishes TLR signaling expands the knowledge of pathogenic variants causing human IRAK4 deficiency.

## METHODS

### Sequencing

A targeted primary immunodeficiency panel was performed for the proband by a commercial service provider (Blueprint Genetics). A detailed sequencing coverage report is provided (Supplemental Table S1).

### Immunoblot

The primary antibodies for immunoblotting detection of human IRAK4 (Lys41; #4363, 1:1000) and β-actin (#3700) were from Cell Signaling Technology. The secondary antibodies conjugated with infrared dye were from LI-COR Bioscience (IRDye 680RD #925-68070, 1:20,000; 800CW #926-32211, 1:20,000). All antibodies were validated as described by the manufacturer. LCLs (B cells) were lysed in modified RIPA lysis buffer (50 mM Tris–HCl, 150 mM NaCl, 2 mM EGTA and EDTA, and 1% Triton X-100, pH 7.5) supplemented with Halt protease and phosphatase inhibitor (Thermo Scientific). Proteins were separated by sodium dodecyl sulfate (SDS)–polyacrylamide gel electrophoresis (PAGE) (10%), and then transferred onto a polyvinylidine fluoride (PVDF) membrane (Immobilon-FL, EMD Millipore). After blocking for 30 min, primary antibodies were applied overnight at 4°C. The membranes were then incubated with secondary antibodies for 1 h at room temperature. A LI-COR Odyssey infrared imager (LI-COR Bioscience) was used for imaging, and densitometry was performed using ImageJ (NIH).

### Cytometric Bead Array (CBA)

LCLs were suspended in supplemented RPMI media and were dispensed into a flat-bottom 24-well plate (2 × 10^6^/well in 1 mL). Control and patient LCLs were stimulated with 4 μg/mL imiquimod (IMQ, InvivoGen), or 50 ng/mL PMA (Sigma-Aldrich) plus 1 μM ionomycin (Sigma-Aldrich) (positive control), or medium alone (negative control). All stimuli were assayed in duplicate. The cells were incubated at 37°C in a 5% CO_2_ atmosphere for 24 h before supernatants were harvested. A CBA was used to measure the concentration of TNF-α (BD Biosciences), performed as previously described (Carson and Vignali 1999). Samples were acquired using a BD LSRFortessa flow cytometer and analyzed using FlowJo.

## ADDITIONAL INFORMATION

### Database Deposition and Access

The variant has been submitted to ClinVar (https://www.ncbi.nlm.nih.gov/clinvar) under accession number SCV001194323. Raw sequencing data was not deposited because of lack of patient consent.

### Ethics Statement

All study participants and/or their parents/guardians provided written informed consent. Research study protocols were approved by The University of British Columbia Research Ethics Board (H15-00641).

### Acknowledgments

We thank the patient and his family for their participation in this research study.

### Author Contributions

A.J. contributed to the pedigree and functional experimental analysis, variant interpretation, and summary. E.J. contributed to case presentation, clinical and genetic findings, and summary. M.S. and H.Y.L. contributed to the laboratory workup. B.P.M. contributed to the technical genetic analysis, ACMG classification, and revisions. C.M.B., K.J.H., S.E., H.K., and A.C. contributed to the diagnosis and clinical management of the patient and family. S.E.T. secured funding, supervised clinical and laboratory work, and wrote the manuscript in partnership with E.J. and A.J. All authors have approved the current version of the manuscript and its submission to *CSH Molecular Case Studies*.

### Funding

This work was supported by Genome British Columbia-SIP007. S.E.T. is supported by a Tier 1 Canada Research Chair in Pediatric Precision Health.

### Competing Interest Statement

The authors have declared no competing interest.

### Referees

Dustin Baldridge

Jim Connelly

## Supplementary Material

Supplemental Material
