# Supplementary material for: Clinical IRAK4 deficiency caused by homozygosity for the novel IRAK4 (c.1049delG, p.Gly350Glufs*15) variant
Source: Cold Spring Harb Mol Case Stud. 2020 Jun;6(3):a005298. doi: 10.1101/mcs.a005298 (PMC7304365; doi:10.1101/mcs.a005298)
Supplement: Supplemental Material [file supp_6_3_a005298__index.html]

Supplemental Material 

# Clinical IRAK4 deficiency caused by homozygosity for the novel *IRAK4* (c.1049delG, p.Gly350Glufs\*15) variant

## Supplemental Material

- Supplemental\_Table\_1.docx
