# Supplementary material for: Clinical IRAK4 deficiency caused by homozygosity for the novel IRAK4 (c.1049delG, p.Gly350Glufs*15) variant
Source: Cold Spring Harb Mol Case Stud. 2020 Jun;6(3):a005298. doi: 10.1101/mcs.a005298 (PMC7304365; doi:10.1101/mcs.a005298)
Supplement: Supplemental Material [file supp_mcs.a005298_Supplemental_Table_1.docx]

**Supplementary Table**

| **Service Provider** | **Panel** | **Genes** | **Bases** | **Bases >20X** | **Median Coverage** | **Percent > 20X** |
| --- | --- | --- | --- | --- | --- | --- |
| Blueprint Genetics | Primary Immunodeficiency Panel | 274 | 707898 | 707412 | 161 | 99.93 |

**Table S1**. Sequencing coverage report for the proband adapted from the Blueprint Genetics Report.
